# Predicting one-year mortality of critically ill patients with early acute kidney injury: data from the prospective multicenter FINNAKI study

**DOI:** 10.1186/s13054-015-0848-2

**Published:** 2015-03-27

**Authors:** Meri Poukkanen, Suvi T Vaara, Matti Reinikainen, Tuomas Selander, Sara Nisula, Sari Karlsson, Ilkka Parviainen, Juha Koskenkari, Ville Pettilä, the FINNAKI Study Group

**Affiliations:** Department of Anaesthesia and Intensive Care, Lapland Central Hospital, PL 8041, Ounasrinteentie 22, Rovaniemi, 96 101 Finland; Intensive Care Units, Division of Anaesthesia and Intensive Care Medicine, Department of Surgery, Helsinki University Central Hospital, Haartmaninkatu 4, Helsinki, 00 029 Finland; Department of Anaesthesiology and Intensive Care, North Karelia Central Hospital, Tikkamäentie 16, Joensuu, 80 210 Finland; Science Service Center, Kuopio University Hospital and Kuopio University, Puijonlaaksontie 2, Kuopio, 70 210 Finland; Department of Intensive Care Medicine, Tampere University Hospital, PL 2000, Tampere, 33 521 Finland; Department of Intensive Care, Kuopio University Hospital, Puijonlaaksontie 2, Kuopio, 70 210 Finland; Department of Anaesthesiology, Division of Intensive Care, Oulu University Hospital and Medical Research Center Oulu, Kajaanintie 50, Oulu, 90 220 Finland

## Abstract

**Introduction:**

No predictive models for long-term mortality in critically ill patients with acute kidney injury (AKI) exist. We aimed to develop and validate two predictive models for one-year mortality in patients with AKI based on data (1) on intensive care unit (ICU) admission and (2) on the third day (D3) in the ICU.

**Methods:**

This substudy of the FINNAKI study comprised 774 patients with early AKI (diagnosed within 24 hours of ICU admission). We selected predictors *a priori* based on previous studies, clinical judgment, and differences between one-year survivors and non-survivors in patients with AKI. We validated the models internally with bootstrapping.

**Results:**

Of 774 patients, 308 (39.8%, 95% confidence interval (CI) 36.3 to 43.3) died during one year. Predictors of one-year mortality on admission were: advanced age, diminished premorbid functional performance, co-morbidities, emergency admission, and resuscitation or hypotension preceding ICU admission. The area under the receiver operating characteristic curve (AUC) (95% CI) for the admission model was 0.76 (0.72 to 0.79) and the mean bootstrap-adjusted AUC 0.75 (0.74 to 0.75). Advanced age, need for mechanical ventilation on D3, number of co-morbidities, higher modified SAPS II score, the highest bilirubin value by D3, and the lowest base excess value on D3 remained predictors of one-year mortality on D3. The AUC (95% CI) for the D3 model was 0.80 (0.75 to 0.85) and by bootstrapping 0.79 (0.77 to 0.80).

**Conclusions:**

The prognostic performance of the admission data-based model was acceptable, but not good. The D3 model for one-year mortality performed fairly well in patients with early AKI.

**Electronic supplementary material:**

The online version of this article (doi:10.1186/s13054-015-0848-2) contains supplementary material, which is available to authorized users.

## Introduction

Predictive models for mortality provide an estimate of the probability of death in a particular group of patients. Although they cannot replace clinical judgment in decision-making, they are valuable complementary tools as they can give an objective medical research-based assessment of the severity of illness and the associated risk of death [1]. A combination of a physician’s clinical evaluation with a risk estimate given by a predictive model has a better ability to distinguish patients with high or low probabilities of survival than estimates based solely on prediction of a physician or a model [2]. A reliable assessment of the expected course of the disease is essential both for informing patients and their families about the situation and for clinical decision-making, and also when estimating the potential futility of care. In addition, prediction models are useful in benchmarking, that is, comparing the performance of intensive care units (ICUs).

Acute kidney injury (AKI) is a syndrome that affects a marked proportion of critically ill patients [3-5] and is associated with high consumption of healthcare resources, particularly when renal replacement therapy (RRT) is administered [6-9]. AKI is associated with 90-day mortality up to 34% [3] and an increased mortality attributable to AKI persists up to 10 years after hospital discharge [6,9-15]. Even mild AKI is associated with markedly increased long-term mortality [16]. AKI-specific predictive models are needed to identify patients with AKI and with poor outcome.

Models for predicting mortality, such as Acute Physiology and Chronic Health Evaluation (APACHE) [17,18] and Simplified Acute Physiology Score (SAPS) [19,20], have shown only poor to moderate predictive performance in patients with AKI [21,22]. AKI-specific scoring systems have been developed for prediction of hospital [23-26] or 60-day mortality [27-29]. Some of these models are restricted to RRT-treated patients [10,29]. None of these AKI-specific scores have used any of the modern definitions for AKI; Risk, Injury, Failure, Loss of kidney function, and End-stage kidney disease (RIFLE) [30], Acute Kidney Injury Network (AKIN) [31], or Kidney Disease: Improving Global Outcomes (KDIGO) criteria [32]. Most importantly, these AKI-specific scoring systems have not shown adequate discrimination or calibration ability possibly due to significant differences in case mix, advancements in ICU care, and changes in AKI definition over years [21,22]. However, the predictive power of AKI-specific models has been shown to improve with increasing surveillance time [28].

One-year mortality is a relevant patient-centred outcome compared to discharge policy-influenced hospital mortality [33]. Accordingly, we aimed to develop and validate predictive models for one-year mortality of critically ill patients with AKI using data available on ICU admission and another using data collected by the third day of ICU treatment.

## Methods

### Study patients

We included consecutive patients with early AKI from the Finnish Acute Kidney Injury (FINNAKI) study [3]. The FINNAKI study was conducted in 17 Finnish ICUs between 1 September 2011 and 1 February 2012 [3]. All patients with an emergency admission or with elective postoperative admission with ICU stay expected to exceed 24 hours were enrolled. The following patients were excluded: (1) patients under 18 years of age, (2) intermediate care patients, (3) patients with chronic dialysis, (4) earlier included patients receiving RRT during the previous admission, (5) patients transferred from another study ICU, who were already included in the study for five days, (6) patients without sufficient language skills or without permanent residency in Finland, and (7) organ donors. In the present study we further excluded: (1) patients with AKI diagnosed later than 24 hours after ICU admission, (2) all patients undergoing cardiac surgery due to organizational differences in postoperative care between the participating hospitals, and (3) all patients in one ICU with incomplete data of vasoactive treatment. For patients with multiple admissions, we included the first admission with AKI.

The Ethics committee of the Department of Surgery in Helsinki University Central Hospital approved the FINNAKI study protocol with written, informed consent from patient or proxy and the use of deferred consent. Finnish National Institute of Health approved the collection of data of deceased patients if informed consent could not be obtained.

### Data collection

We prospectively collected data from the first five days in the ICU using the Finnish Intensive Care Consortium database maintained by Tieto Ltd (Helsinki, Finland). The data included admission and outcome data, severity of illness scores, physiological measurements, and laboratory values. Additionally, we used a study-specific case report form (CRF) to collect information regarding predetermined chronic illnesses (chronic obstructive pulmonary disease, hypertension, arteriosclerosis, systolic heart failure, chronic kidney diseases, diabetes mellitus, chronic liver failure, ureterolithiasis, coagulopathies, systemic vasculitis, rheumatoid arthritis, organ transplants and malignancies), daily medications, and predetermined risk factors for AKI (hypotension, resuscitation, hypovolaemia by clinicans’ judgement, transfusion of at least 10 red blood cell units, rhabdomyolysis, acute liver failure, low cardiac output, operation within one week prior to ICU admission, and use of radiocontrast dye/aminoglycosides/pepticoglycans that is vancomycin/angiotensin-converting enzyme inhibitor or angiotensin receptor blocker/non-steroidal anti-inflammatory drugs/amphotericin B/diuretics/metformin/hydroxylethyl starch/gelatin/albumin within 48 hours prior to ICU admission) from patients’ records. The Finnish Population Register Centre provided data regarding vital status at one year from ICU admission (the primary study end point).

### Definitions

We used the KDIGO criteria including both changes in serum creatinine and hourly urine output to define and stage AKI [32]. Early AKI was defined as any AKI stage diagnosed within 24 hours of ICU admission. Chronic kidney disease was defined as glomerular filtration rate <60 ml/ml/1.73 m^2^ for three months [34]. The presence of hypotension (systolic blood pressure <90 mmHg) or resuscitation (haemodynamic collapse requiring cardiopulmonary resuscitation, defibrillation, or administration of epinephrine) within 48 hours preceding ICU admission, organ dysfunctions, and severe sepsis (defined according to the American College of Chest Physicians/Society of Critical Care Medicine criteria [35]) were evaluated by attending physicians. We defined organ failure (OF) as a daily organ-specific Sequential Organ Failure Assessment (SOFA) score >2 [36,37]. The ∆OF was defined as the difference in the number of failed organs on day 3 (D3) versus day 1 (D1) in the ICU [38]. The premorbid functional performance preceding the acute illness was dichotomised: (1) normal or unable to work but no need for assistance in self-care and daily living and (2) some assistance required or totally dependent on assistance.

### Model development

We generated models for one-year mortality using data available at two clinically relevant time points: first, data available on ICU admission (admission model) and, second, on D3 in the ICU (D3 model). We selected candidate predictors for one-year mortality *a priori* based on previous studies, clinical judgment as recommended [1], and by differences in univariable analyses between one-year survivors and non-survivors in this study. The admission model comprised only predictors available at the time of ICU admission to avoid the possible confounding effect of treatment in the ICU. Age, gender, co-morbidities, premorbid functional performance, type of admission according to SAPS II (scheduled surgical, unscheduled surgical, or medical), APACHE II diagnostic group, and presence/absence of severe sepsis were included in both models. The admission model also included data on presence of hypotension or resuscitation preceding ICU admission. In the D3 model, we included organ-supportive treatments (mechanical ventilation and administration of norepinephrine) on D3 in the ICU, change in the number of registered organ failures including renal failure (∆OF), the worst value of base excess (BE) and platelets on the D3 in the ICU, and highest bilirubin concentration during the first three days in the ICU, and SAPS II points without points for age, chronic health status, admission type, and renal components. We studied the potential existence of collinearity with multiplicative terms.

First, we analysed the association between candidate predictors and one-year mortality with multivariable logistic regression analysis separately for both admission and D3 models. We performed backwards elimination at the significance level of *P* <0.05. We assessed discrimination (the model’s ability to distinguish survivors from non-survivors) by concordance statistic (c-statistic) using the area under the receiver operating characteristic curve (AUC). We regarded AUC values of >0.7, >0.8, and >0.9 as acceptable, good, or excellent, respectively [39]. We evaluated calibration (the model’s ability to accurately predict the number of deaths across different levels of risk) with the Hosmer-Lemeshow goodness-of fit (GoF) test with *P* value >0.05 indicating good calibration [40]. We assessed the overall predictive accuracy with the Brier score [40], which is the mean squared difference between the observed outcome and predicted risk of death. A low Brier score indicates good model performance (0 for a perfect model). When mortality is 50%, a Brier score of 0.25 would indicate that the model is worthless; for a mortality of 40%, the upper limit of the Brier score is 0.24 [41]. Finally, we calculated the standardized mortality ratios (SMRs) with both models by dividing the number of observed deaths with the predicted number of deaths.

If no data regarding pre-existing chronic illnesses were recorded, we assumed that particular condition not to exist [42]. For continuous data we substituted missing values by the median value of the variable. The proportion of missing data on candidate predictors ranged from 0 to 2.6% in the admission model and from 0 to 6.0% in the D3 model.

For comparison, we determined the discrimination of the SAPS II score when it was used alone as a predictor of one-year mortality.

### Model validation

We validated the models internally by bootstrapping. We performed a bootstrap procedure (1,000 draws with replacement) to obtain the bootstrap-adjusted c-statistic index. The bootstrap method randomly draws multiple samples with replacement from the original cohort. The model is developed again in each bootstrap sample yielding a different AUC (c-statistic) to each bootstrap model [40]. Finally, an average of these c-indexes was calculated.

### Statistical analysis

We report categorical variables as absolute numbers with percentages and continuous data as median with interquartile ranges (IQRs). Categorical data were compared using the chi-square or Fisher’s exact test when appropriate. We used the Mann-Whitney *U* test to compare continuous data. We calculated the independent contribution of each variable included in the prediction model by dividing the difference in the −2 log likelihood of the null model and the final model without the particular predictor by the difference in the −2 log likelihood of the null model and the complete final model [43]. This ratio was normalised to presents. Statistical analyses were performed using SPSS Statistics version 20 software (IBM, Armonk, NY, USA) and R version 3.0.3 for Mac (R Foundation for Statistical Computing, Vienna, Austria).

## Results

### Patients

**Figure 1:**
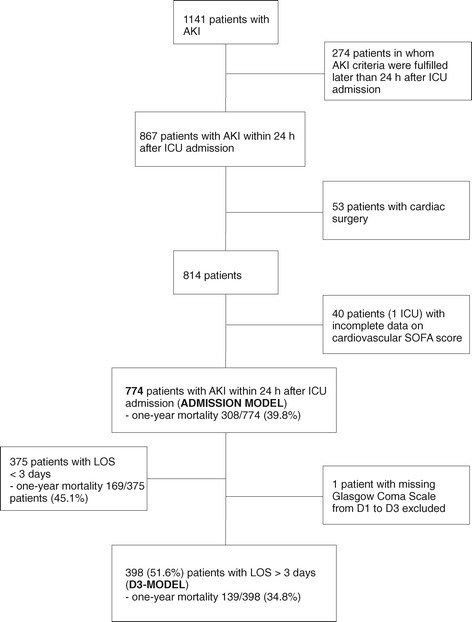
**Study flow chart.** AKI, acute kidney injury; D1/D3, the first/third day in the ICU; ICU, intensive care unit; LOS, length of stay; SOFA, Sequential Organ Failure Assessment.

Of the 774 critically ill patients with early AKI (AKI diagnosed within 24 hours of ICU admission), 399 (51.6%) stayed in the ICU for at least three days. We excluded 274 patients (24% of all 1,141 AKI patients) with AKI occurring later than 24 hours after admission. Figure 1 illustrates the study flow chart. The overall one-year mortality rate for patients with early AKI was 308/774 (39.8%, 95% confidence interval (CI) 36.3 to 43.3%). Of the 308 patients who died within one year, 222 (72%) died in the hospital. The Kaplan-Meier one-year survival plot is shown in Additional file 1. Table 1 presents the characteristics of one-year survivors and non-survivors. No differences in the proportion of patients with severe sepsis, use of RRT in ICU, or in severity of AKI between one-year survivors and non-survivors existed. Treatment restrictions were applied in 202/774 (26.1%) patients.

**Table 1:** **Characteristics of the one-year survivors and non-survivors among critically ill patients with acute kidney injury within 24 hours of ICU admission**

	**Data**	**Survivors n = 466**	**Data**	**Non-survivors n = 308**	***P***
Age, years	466	62.0 (53.0-72.0)	308	70.0 (61.0-79.0)	<0.001
Gender, male	466	307 (65.9)	308	199 (64.6)	0.716
Body mass index, kg/m^2^	465	27.8 (24.3-32.3)	306	26.5 (23.9-30.5)	<0.001
Hypertension	465	249 (53.5)	306	175 (57.2)	0.320
Systolic heart failure	460	39 (8.5)	300	58 (19.3)	<0.001
Arteriosclerosis	462	58 (12.6)	303	62 (20.5)	0.003
Chronic obstructive pulmonary disease	463	39 (8.4)	304	36 (11.8)	0.119
Diabetes mellitus	466	132 (28.3)	307	71 (23.1)	0.108
Malignancy	462	40 (8.7)	303	60 (19.8)	<0.001
Chronic kidney disease	463	37 (8.0)	306	43 (14.1)	0.007
Chronic liver disease	463	17 (3.6)	301	27 (9.0)	0.002
Immunosuppression	442	20 (4.5)	288	28 (9.7)	0.006
Number of co-morbidities	466	1.0 (0.0-2.0)	308	2.0 (1.0-3.0)	<0.001
0 co-morbidity	466	146 (31.3)	308	50 (16.2)	<0.001
1-2 co-morbidities	466	223 (47.9)	308	154 (50.0)	0.559
≥3 co-morbidities	466	97 (20.8)	308	104 (33.8)	<0.001
Premorbid functional performance preceding the acute illness					<0.001
Normal or disabled to work but no need for assistance	465	405 (87.1)	308	238 (77.3)	<0.001
Some assistance required or totally dependent on assistance	465	60 (12.9)	308	70 (22.7)	<0.009
Prior to ICU admission					
Hypotension prior to ICU admission^a^	454	154 (33.9)	300	150 (50.0)	<0.001
Resuscitation prior to ICU admission^b^	466	34 (7.3)	305	55 (18.0)	<0.001
Lactate prior to ICU admission, mmol/l	308	2.8 (1.5-5.3)	191	3.6 (1.5-8.1)	0.027
pH prior to ICU admission	369	7.30 (7.19-7.38)	224	7.27 (7.14-7.36)	0.111
Base excess prior to ICU admission, mmol/l	366	−6.7 (−12.5-(−2.5))	221	−7.8 (−14.4-(−3.5))	0.084
Creatinine prior to ICU admission, μmol/l	431	136.0 (82.0-254.0)	285	141.0 (90.0-220.0)	0.794
Platelets prior to ICU admission, E^9^/l	437	209.0 (141.5-282.5)	284	175.5 (101.3-266.8)	0.001
Admission type according to SAPS II					
Scheduled surgical	466	23 (4.9)	308	2 (0.6)	0.001
Unscheduled surgical	466	126 (27.0)	308	65 (21.1)	0.061
Medical	466	317 (68.0)	308	241 (78.2)	0.002
SAPS II, points	466	41.0 (32.0-51.0)	308	60.0 (47.0-73.0)	<0.001
SOFA D1, points	466	8.0 (6.0-11.0)	308	11.0 (9.0-14.0)	<0.001
SOFA D3, points	259	7.0 (1.0-14.0)	138	9.0 (2.48-17.0)	<0.001
Number of OF on D3^c^	255	1.0 (0.0-3.0)	138	2.0 (0.0-4.5)	<0.001
0-1 OF^c^	255	174 (66.9)	138	65 (47.1)	<0.001
2 OF^c^	255	61 (23.5)	138	39 (28.3)	0.29
3 OF^c^	255	18 (6.9)	138	24 (17.4)	0.001
≥4 OF^c^	255	(0.8)	138	10 (7.2)	<0.001
∆OF^c,d^	255	−1.0 (−3.0-1.0)	138	0 (−3.0-1.5)	0.068
During the first 3 ICU days					
KDIGO stage 1	466	177 (38.0)	308	96 (31.2)	0.052
KDIGO stage 2	466	110 (23.6)	308	70 (22.7)	0.777
KDIGO stage 3 without RRT	466	64 (13.7)	308	49 (15.9)	0.402
RRT	466	115 (24.7)	308	93 (30.2)	0.090
Mechanical ventilation	466	293 (62.9)	308	248 (80.5)	<0.001
Severe sepsis	466	184 (39.5)	308	142 (46.1)	0.068
Length of stay (days)					
ICU	466	3.4 (1.9-5.8)	308	2.7 (1.1-5.9)	0.001
Hospital	466	13.0 (8.0-23.0)	308	8.0 (3.0-18.8)	<0.001

^a^Hypotension was defined as systolic blood pressure <90 mmHg for 1 hour within 48 hours prior to ICU admission; ^b^resuscitation was defined as haemodynamic collapse requiring cardiopulmonary resuscitation, defibrillation or administration of epinephrine within 48 hours prior to ICU admission; ^c^patients with ICU stay for at least 3 days (n = 398); ^d^∆OF was defined as the difference in the number of organ failures on D3 versus D1. ICU, intensive care unit; SAPS, Simplified Acute Physiology Score; SOFA, Sequential Organ Failure Assessment; KDIGO, Kidney Disease: Improving Global Outcomes; RRT, renal replacement therapy; D1/D3 the first or third day on the ICU.

### Admission model

The admission model comprised 774 patients. Table 2 presents the results of the regression analysis with odds ratios (OR, 95% CI) of all significant predictors. Discrimination of the model was acceptable with an AUC (95% CI) of 0.76 (0.72 to 0.79). The model was well calibrated (Hosmer-Lemeshow GoF 10.35, *P* = 0.24). The mean bootstrap-adjusted AUC of the admission model was 0.75 (95% CI 0.74 to 0.75). The Brier score yielded a value of 0.20 (95% CI 0.20 to 0.20). The logistic regression equation and probability of death with two patient examples are presented in Additional file 2. The bootstrap-adjusted mean SMR (95% CI) was 1.00 (0.92 to 1.09). Altogether 29 patients (3.7%) had a probability of death within one year over 80%, and 24 of those 29 (82.8%) died. Their characteristics are presented in Table 3.

**Table 2:** **Admission model for one-year mortality by multivariate logistic regression analysis**

	**Odds ratio (95% CI)**	***P***	**Independent contribution %** ^**a**^
Age, years (per year)	1.03 (1.02-1.04)	<0.001	15.3
Admission type according to SAPS II^b^			11.7
Unscheduled surgical	7.74 (1.65-36.23)	0.009	
Medical	11.30 (2.45-52.01)	0.002	
Chronic liver failure	3.79 (1.89-7.43)	<0.001	10.3
Malignancy	2.34 (1.43-3.83)	0.001	9.4
Resuscitation prior to ICU admission^c^	2.34 (1.42-3.85)	0.001	8.9
Dependence of assistance in premorbid functional performance preceding the acute illness^d^	1.75 (1.15-2.68)	0.009	8.7
Hypotension prior to ICU admission^e^	1.67 (1.20-2.31)	0.002	8.3
Arteriosclerosis	1.87 (1.19-2.95)	0.007	7.3
Diabetes mellitus	0.59 (0.41-0.86)	0.006	7.3
Systolic heart failure	1.83 (1.13-2.95)	0.014	6.8
Immunosuppression	1.97 (1.00-3.90)	0.052	5.7

^a^Presents the independent contribution percentage of the variable to the predictive performance of the model; ^b^compared to scheduled surgical; ^c^resuscitation was defined as haemodynamic collapse requiring cardiopulmonary resuscitation, defibrillation or administration of epinephrine within 48 hours prior to ICU admission; ^d^compared to normal or disable to work; ^e^hypotension was defined as systolic blood pressure <90 mmHg for 1 hour within 48 hours prior to ICU admission. Non-significant predictors for one-year mortality included in the analysis: gender, body mass index, APACHE II (Acute Physiology and Chronic Health Evaluation) diagnostic group, co-morbidities (hypertension, chronic obstructive pulmonary disease), glomerular filtration rate, operation within a week prior to ICU admission, and severe sepsis 24 h prior to ICU. CI, confidence interval; SAPS, Simplified Acute Physiology Score; ICU, intensive care unit.

**Table 3:** **Characteristics of patients with over 80% risk of death within one year according to the admission model**

	**ADM model (n = 29)**
Age (median, IQR)	72 (67-81)
Dependence of assistance in premorbid functional performance preceding the acute illness	12 (41.4%)
Admission type according to SAPS II	
Unscheduled surgical	1 (3.4)
Medical	28 (96.6)
Arteriosclerosis	11 (37.9)
Systolic heart failure	11 (37.9)
Chronic liver failure	8 (27.6)
Diabetes mellitus	4 (13.8)
Malignancy	15 (51.7)
Immunosuppression	10 (34.5)
Hypotension prior to ICU admission^a^	21 (72.4)
Resuscitation prior to ICU admission^b^	16 (55.2)

^a^Hypotension was defined as systolic blood pressure <90 mmHg for 1 hour; ^b^resuscitation was defined as haemodynamic collapse requiring cardiopulmonary resuscitation, defibrillation or administration of epinephrine. ADM, admission model; IQR, interquartile range; SAPS, Simplified Acute Physiology Score; ICU, intensive care unit.

In a subgroup of hospital survivors (552 patients), the predictive ability of the admission model for the one year mortality yielded an AUC value of 0.70 (0.64 to 0.76) with GoF 12.95, *P* = 0.11). Likewise, the performance of the admission model to predict hospital mortality was acceptable with an AUC (95% CI) of 0.76 (0.72 to 0.79) and calibration of 7.17 by GoF, *P* = 0.52.

### D3 model

**Figure 2:**
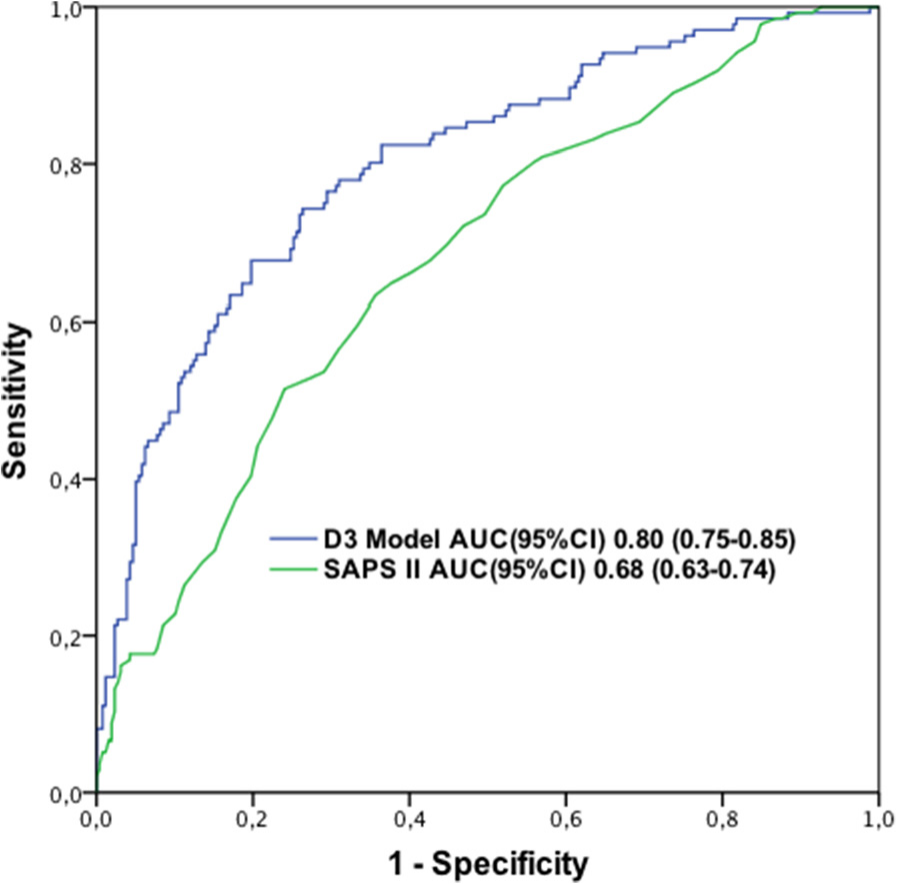
**Discrimination of the D3 model and SAPS II for one-year mortality by the area under the receiver operating characteristic curve (AUC).** D3 the third day on the ICU; ICU, intensive care unit; SAPS, Simplified Acute Physiology Score.

The D3 model comprised 398 patients. Of these, 138 were dead by one year (34.7%, 95% CI 29.9 to 39.4%). Table 4 presents the D3 model. Figure 2 illustrates the ROC curve of the D3 model. The D3 model performed well with an AUC (95% CI) of 0.80 (0.75 to 0.85) and calibration of 7.70 by GoF, *P* = 0.46. The bootstrapped mean AUC was 0.79 (95% CI 0.77 to 0.80). The Brier score yielded a value of 0.17 (95% CI 0.17 to 0.18). The logistic regression equation and calculation of the probability of death are shown in an Additional file 2. The mean SMR (95% CI) of the bootstrapped models was 1.0 (0.89 to 1.13). Nineteen patients (4.8%) had a probability over 80% of death and 16 (84.2%) of them died within one year. All these high-risk patients were medical admissions, four (21.2%) required assistance in their daily living, and they had a median (IQR) of two (one to three) co-morbidities. Their median (IQR) age was 68 (51 to 78) years, modified SAPS II score 34 (12 to 42), BE on D3 -4.7 (-7.6 to -2.1) mmol/l, and maximum bilirubin value by D3 102 (29 to 168) μmol/l. Eighteen (94.7%) still required mechanical ventilation on D3. Additional file 2 presents a patient example.

**Table 4:** **Day 3 model for one-year mortality by multivariate logistic regression analysis**

**Predictor**	**Odds ratio (95% CI)**	***P***	**Independent contribution %** ^**a**^
The highest bilirubin within D1 to D3 (per μmol/l)^b^	1.02 (1.01-1.03)	<0.001	22.6
Age, years (per year)	1.04 (1.02-1.06)	<0.001	15.6
Mechanical ventilation on D3	2.73 (1.62-4.61)	<0.001	15.0
SAPS II score without points given for age, renal components, bilirubin, and type of admission (per point)	1.03 (1.00-1.05)	0.02	12.3
The lowest BE value on D3 (per mmol/l)	0.92 (0.87-0.97)	0.001	11.8
Number of co-morbidities	1.26 (1.05-1.52)	0.015	8.0
Dependence of assistance in premorbid functional performance preceding the acute illness^c^	1.76 (0.91-3.42)	0.094	5.9
Admission type according to SAPS II^d^			8.9
Unscheduled surgical	3.97 (0.46-33.98)	0.208	
Medical	6.59 (0.79-55.05)	0.08	

^a^Presents the independent contribution percentage of the variable to the predictive performance of the model; ^b^the proportion of missing values of the bilirubin concentration within the first three days was 6.0%. ^c^compared to normal or unable to work; ^d^compared to scheduled surgical. Non-significant predictors for one-year mortality included to the analysis: APACHE II diagnostic group, gender, daily performance, admission type according to SAPS II, severe sepsis between admission and D3, use of norepinephrine on D3, the lowest platelet value on the D3, the highest Kidney Disease: Improving Global Outcomes (KDIGO) stage during D1 to D3, and difference in number of organ failures, including renal failure, on D3 and D1 (ΔOF)). CI, confidence interval; D1/D3 the first or third day on the ICU; SAPS, Simplified Acute Physiology Score; BE, base excess; ICU, intensive care unit.

Among hospital survivors treated in the ICU for at least three days (312 patients) the ability of the model to predict one-year mortality yielded AUC of 0.69 (0.61 to 0.76) and calibration of 7.74 by GoF, *P* = 0.46.

### SAPS II score

The AUC (95% CI) of the SAPS II score alone as a predictor of one-year mortality was 0.68 (0.63 to 0.74).

## Discussion

In this follow-up study of the prospective FINNAKI study, we sought to identify factors predicting one-year mortality of critically ill patients with early AKI and to construct predictive models for one-year mortality using data available on ICU admission, and separately, on D3 in the ICU. Severity of the acute illness, advanced age, diminished premorbid functional performance, a higher number of co-morbidities, as well as need for mechanical ventilation on D3 were independent predictors of poor long-term outcome. In contrast, neither the severity of AKI nor the presence of severe sepsis was independently associated with one-year mortality. The prognostic performance of the admission data-based model was acceptable but not good. The discrimination improved with a longer surveillance period and the performance of the D3 model was fairly good with an AUC value of 0.8. Recently, some authors have even considered an AUC of 0.8 excellent [44]. The SAPS II score alone was a poor predictor of long-term outcome.

To the best of our knowledge, the current study is the first to report models for prediction of one-year mortality in non-selected patients with early AKI. Moreover, these models outperform the majority of previously developed models. Of previous AKI-specific scores, Liano *et al*.’s score [26] performed poorly, while the SHARF score performed fairly well with an AUC 0.8 [12]. These models were tested among hospital survivors of the SHARF score development cohort, and consequently, good performance of the SHARF score was to be expected. Chertow *et al*. have developed predictive models for 60-day mortality in patients with AKI of on three separate time points (day of diagnosing AKI, day of consultation of a nephrologist, and day of initiation of RRT) [28]. Although the performance of these models improved over time, the discrimination was from poor to moderate (AUC values of 0.62, 0.68 and 0.72, respectively) [28]. Among patients receiving RRT, predictive models have performed better with AUCs from 0.83 [10] to 0.85 [29]. As RRT-treated patients form a homogenous patient cohort, the performances of the models were presumably better.

At ICU admission, the three strongest predictors of death within one year were (1) advanced age, (2) type of ICU admission (medical or emergency surgical admission), and (3) underlying chronic liver failure contributing approximately 37% to the model. An emergency admission has been associated with unfavourable prognosis in several other models [17,19,29]. As in our model, the presence of several co-morbidities has been associated with one-year mortality among patients with severe AKI [10] and also with short-term mortality in patients with AKI [23-29]. Additionally, we found the presence of malignancy, need for resuscitation pre-ICU, and need for assistance in daily living to be strong predictors for one-year mortality (see Table 2). These findings corroborate a recent study that found frailty among older general ICU patients to predict six-month mortality [45]. Interestingly, diabetes was found to be a predictor for better long-term survival in the present study. A total of 203 (26.2%) of the 774 patients with early AKI had diabetes. In the FINNAKI study, 22.0% of the critically ill patients had diabetes and a higher proportion of diabetes was found in patients with AKI (26.6%) than in those without AKI (19.6%) [3]. However, the proportion of diabetics on medication is much lower (4.5%) in the general Finnish population [46,47]. Thus, diabetics were relatively overrepresented in our study population. It thus seems that diabetes predisposes to severe acute illnesses and acute kidney injury. Nevertheless, in this population of ICU patients with AKI, diabetes was associated with improved outcome. Based on our data, we are not able to give any definitive explanation for this finding. However, the finding is somewhat similar to the ‘obesity paradox’: obesity increases the susceptibility for AKI, but is associated with improved survival among patients with RRT-treated severe AKI [48].

Most of the previous AKI-specific models are based on data available at the time of diagnosing AKI. A worse outcome has been found in patients with gradually progressing AKI compared to patients with stable AKI or improving AKI [49]. In line, worsening of organ function over the first three days after initiation of RRT or three to four days after the onset of sepsis has been shown to associate with decreased survival [38,50,51]. Therefore, scores based solely on the data within the first day of ICU or at the time of diagnosing AKI may not be reliable in predicting the outcome of patients with progressive critical illness. In addition, previous studies have shown the predictive performance of models among AKI patients to improve over time [23,28]. Thus, we generated another model using data available by D3 in the ICU. In line with other AKI-specific models, we found that (1) advanced age, (2) need for mechanical ventilation, and (3) hepatic dysfunction (the highest bilirubin value) were the strongest predictors of long-term mortality [23-26,28,29] contributing to 51% of the D3 model (see Table 4). Of patients with a probability of over 80% of dying, 95% needed mechanical ventilation on D3. Besides advanced age, also the lowest base excess value on D3 reflecting the severity of metabolic acidosis of varying aetiology was a predictor of worse outcome. Thus, among patients with early AKI, other organ failures on D3 that have been refractory to ICU treatment or have developed while in ICU, serve as predictors of adverse long-term outcome.

Interestingly, we did not find the presence of severe sepsis to associate with one-year mortality. This finding is in contrast to two AKI-specific models based on data from years 1997 to 1998 [24] and 1999 to 2001 [28] that have included the presence of severe sepsis as a predictor of adverse outcome. Recent studies have reported reduced mortality in severe sepsis suggesting a beneficial effect of increased knowledge, better recognition, and earlier treatment of these patients [52,53]. This improved performance in the treatment of septic patients may explain why severe sepsis did not remain as an independent predictor in our models. Correspondingly, the increased awareness and advances in the treatment of AKI [30-32] may account for the somewhat unexpected finding that severity of AKI was not an independent predictor of one-year mortality.

A model with good performance for short-term mortality also predicts long-term mortality well if most deaths occur in the hospital. In the present study, the majority of the deaths (72%) occurred in the hospital. This partly explains that the admission model predicted hospital and one-year mortality equally well. Among hospital survivors treated in the ICU for at least three days, the D3 model performed worse (AUC 0.69) than it did in the overall population, suggesting that the discrimination ability of the D3 diminishes after hospital discharge.

Our study has several strengths. The participating ICUs represent both academic and non-academic ICUs, and their referral areas cover 85% of the Finnish adult population [3]. All data were recorded prospectively. We defined and staged AKI by changes in both serum creatinine and hourly urine output with the latest AKI definition [32]. The models comprised data that were routinely collected during ICU stay and are easily repeatable. However, several limitations need to be discussed. First, we selected candidate predictors based on previous studies and clinical judgement. Some significant factors may have been excluded from the analysis due to low frequency of the variable or large proportion of missing data (we avoided including variables with >5% of missing data) [54]. In addition, we had to exclude 4.9% of AKI patients with missing reliable cardiovascular SOFA score (Figure 1). Second, our study was not multinational, which may limit the generalizability of the results. However, the predictors of adverse outcome found in the D3 model were in line with the previous AKI-specific models [23-29]. In addition, the mean bootstrap-adjusted SMRs of both models support the validity of models. Third, our models include rather many predictor variables, which may increase the risk for overfitting. However, the large simulation study by Vittinghoff and McCulloch demonstrated that causal influences can generally be adequately analysed even when there are no more than five to nine outcome events per predictor variable [55]. Finally, though we were not able to validate our models externally, we used the bootstrapping technique for validation that is considered as the preferred method for internal validation [40].

## Conclusions

We developed two new predictive models for one-year mortality among critically ill patients with early AKI using data available on ICU admission and on D3. The prognostic performance of the admission model was acceptable, but not good, whereas the performance of the D3 model was fairly good.

## Key messages

- A predictive model for one-year mortality for critically ill patients with early AKI based on data on D3 performed fairly well.
- Severity of illness, advanced age, poor premorbid functional performance, a high number of co-morbidities, and need for mechanical ventilation on D3 were predictors of a poor long-term outcome.
- Severe sepsis and the severity of AKI were not independent predictors.

## Additional files

Additional file 1:
**A PDF file containing one picture.** One-year survival of patients with early AKI. Additional file 2:
**A PDF file containing one table and text.** The logistic regression equations for admission model and D3 model and equation of probability of death.

## Abbreviations

∆OF: difference in the number of failed organs on day 3 versus day 1 in the ICU
AKI: acute kidney injury
AKIN: Acute Kidney Injury Network
APACHE: Acute Physiology and Chronic Health Evaluation
AUC: area under the receiver operating characteristic curve
BE: base excess
CI: confidence interval
CRF: case report form
GoF: goodness of fit
ICU: intensive care unit
IQR: interquartile range
KDIGO: Kidney Disease: Improving Global Outcomes
OF: organ failure
OR: odds ratio
RIFLE: Risk, Injury, Failure, Loss of kidney function, and End-stage kidney disease
RRT: renal replacement therapy
SAPS: Simplified Acute Physiology Score
SMR: standardized mortality ratio
SOFA: Sequential Organ Failure Assessment
