# Supplementary material for: Predicting one-year mortality of critically ill patients with early acute kidney injury: data from the prospective multicenter FINNAKI study
Source: Crit Care. 2015 Mar 27;19(1):125. doi: 10.1186/s13054-015-0848-2 (PMC4407305; doi:10.1186/s13054-015-0848-2)

ADDITIONAL FILE:

Additional file 1. The Kaplan-Meier one-year survival plot of patients with early AKI.

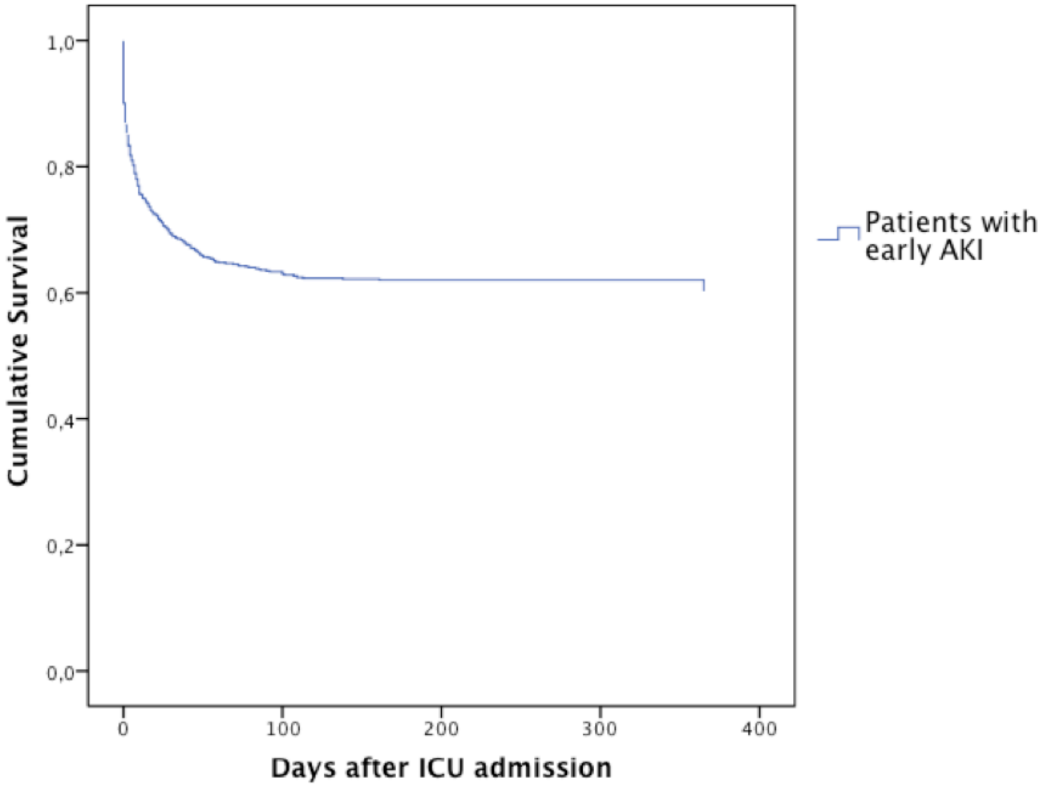

Supplement: Additional file 1: — A PDF file containing one picture. One-year survival of patients with early AKI. [file 13054_2015_848_MOESM1_ESM.pdf]
