# Supplementary material for: Predicting one-year mortality of critically ill patients with early acute kidney injury: data from the prospective multicenter FINNAKI study
Source: Crit Care. 2015 Mar 27;19(1):125. doi: 10.1186/s13054-015-0848-2 (PMC4407305; doi:10.1186/s13054-015-0848-2)
Supplement: Additional file 2: — A PDF file containing one table and text. The logistic regression equations for admission model and D3 model and equation of probability of death. [file 13054_2015_848_MOESM2_ESM.pdf]

ADDITIONAL FILES

Additional file 2

The logistic regression equations for admission model and D3 model and equation of probability of death.

**Admission model**
logit= -5.382 + 0.031 •age (years) + 0.562 •functional performance before acute illness[a] (0/1) + 2.046• unscheduled surgical admission (0/1) + 2.425 • medical admission (0/1) + 0.627 • arteriosclerosis (0/1) + 0.601 • systolic heart failure (0/1) + 1.321 • chronic liver failure (0/1) + (-0.524) • diabetes mellitus (0/1) + 0.852 • malignancy (0/1) + 0.510 • hypotension preceding ICU[b] (0/1) + 0.850 • resuscitation preceding ICU[c] (0/1) + 0.679 • immunosuppression (0/1)
**D3 Model:**
logit = -7.126 + 0.039 • age (years) + 0.566 • functional performance before acute illness[a] + 1.379• unscheduled surgical admission (0/1) + 1.886 • medical admission (0/1) + 0.026 • modified SAPS II score[d] + 0.233• number of co-morbidities + 1.006 • mechanical ventilation (0/1)[e] + (-0.087) • BE (mmol/l)[f] + 0.017 • bilirubin (µmol/l)[g]
**Probability of death** = exp (logit) / [1 + exp (logit)]

[a]Functional performance preceding the acute illness was defined as normal or disability to work but no need for assistance in self-care and daily living (0) and some assistance required or totally dependent on assistance (1).

[b]Hypotension preceding ICU admission was defined systolic blood pressure <90 mmHg for 1 hour; [c] resuscitation as haemodynamic collapse requiring cardiopulmonary resuscitation, defibrillation or administration of epinephrine;

[d] SAPS (Simplified Acute Physiology Score) II points without points for age, chronic health status, admission type, and renal components; [e] mechanical ventilation on the third day in the ICU and [f] the lowest BE value on the third day in the ICU [g] the highest bilirubin concentration by the first three days in the ICU

Patient example 1.

A 70-year-old male with a history of hypertension, systolic heart failure, and chronic obstructive pulmonary disease was admitted from the emergency room (medical admission). He was totally dependent on assistance in his daily functions. He had developed cardiogenic shock and been resuscitated. He had KDIGO AKI stage 2 and his SAPS II score was 93. According to the prediction model, he yielded an 85% probability of death. He died within a few hours in the ICU without treatment restrictions.

Patient example 2.

A 79-year-old male had a history of hypertension, arteriosclerosis, and vasculitis was admitted because of septic shock. He survived his daily living without assistance. His SAPS II score within 24 h of ICU treatment was 52. He developed severe stage 3 AKI. On Day 3, he required mechanical ventilation and his bilirubin was 106µmol/l with increasing

trend. According to the ADM model his probability death was 53% and according to the D3 model 93%. He died after nine days of ICU treatment.
